# Serial Monitoring of Circulating Tumor DNA in Patients With Metastatic Colorectal Cancer to Predict the Therapeutic Response

**DOI:** 10.3389/fgene.2019.00470

**Published:** 2019-05-21

**Authors:** Ning Jia, Zhao Sun, Xin Gao, Yuejuan Cheng, Yanping Zhou, Chunying Shen, Wei Chen, Xueliang Wang, Rong Shi, Nan Li, Jianfeng Zhou, Chunmei Bai

**Affiliations:** ^1^Department of Medical Oncology, Peking Union Medical College Hospital, Chinese Academy of Medical Sciences, Beijing, China; ^2^Department of Radiology, Peking Union Medical College Hospital, Chinese Academy of Medical Sciences, Beijing, China; ^3^San Valley Biotechnology Incorporated, Beijing, China; ^4^Clinical Epidemiology Research Center, Peking University Third Hospital, Beijing, China

**Keywords:** liquid biopsy, circulating tumor DNA, metastatic colorectal cancer, therapeutic response, biomarker

## Abstract

Early biomarkers of therapeutic responses can help optimize the treatment of metastatic colorectal cancers (mCRC). In this prospective exploratory study, we examined serial changes of plasma-circulating tumor DNA (ctDNA) in 41 mCRC patients receiving first-line chemotherapies and tested its association with treatment outcomes according to radiological assessments. Using next-generation sequencing technologies, we profiled somatic mutations in 50 cancer-related genes in ctDNA before each of the first four treatment cycles. We observed mutations in 95.7% of pre-treatment ctDNA samples. Using mutations of the maximal frequency in each pre-treatment plasma ctDNA sample as the candidate targets, we computed log2 fold changes of ctDNA levels between adjacent treatment cycles. We found that ctDNA reductions as early as prior to cycle 2 predicted responses after cycle 4. Log2 fold changes of ctDNA after cycle 1 (ctDNA log2 (C1/C0)) > −0.126 predicted progressive disease, with an accuracy of 94.6%. These patients also showed significantly worse progression-free survival than those with ctDNA log2 (C1/C0) ≤ −0.126 (median 2.0 vs. 9.0 months; *P* = 0.007). Together, the present exploratory study suggests that early changes in ctDNA levels detected via targeted sequencing are potential biomarkers of future treatment responses in mCRCs.

## Highlights

- Mutations in pretreatment ctDNA were detected in 95.7% mCRC via targeted sequencing.
- Early changes in ctDNA could predict future radiologically assessed responses in mCRC.
- Early biomarkers of therapeutic responses could help optimize the treatment.

## Introduction

Colorectal cancer is the third most common cancer in the world, and ranks second in terms of mortality (Bray et al., 2018). Advances in systemic therapies have significantly improved the survival of patients with mCRC (Fakih, 2015; Van Cutsem et al., 2016; McQuade et al., 2017), and new treatment options are emerging in refractory mCRC (Le et al., 2015; Sartore-Bianchi et al., 2016b). However, there is a high variability of therapeutic responses among patients and determining the optimal personalized treatment plan is challenging. Predictive biomarkers of treatment outcomes and disease progression are of great values in clinical decision-making that enables early assessment of treatment responses and choice of alternative therapies to avoid unnecessary side effects, enhance efficacy, and minimize costs.

Conventional monitoring is primarily based on imaging and measurements of traditional serum tumor markers, e.g., CEA and CA19-9, as suggested in the RECIST. However, radiation exposure, costs, logistics and operational constraints limit the frequency with which CT scans can be performed. Therefore, radiological assessments are usually not conducted until about two months after starting treatments, which may delay the selection of alternative therapies for patients unresponsive to first-line treatments. Furthermore, some lesions are immeasurable on CT scans, and new lesions that appear on serial CT scans are commonly due to non-neoplastic causes or a different cancer. The biologic agents that have been widely used in recent years may cause lesions to form tumoral cavities (Chun et al., 2009; Kawasaki et al., 2016), which are signs of efficacy, but the tumor sizes could become even bigger. Serum tumor markers can be assayed more frequently, but their sensitivity and specificity are low (Bast et al., 2001; Goldstein and Mitchell, 2005). Transient therapy-related surges in serum tumor markers, such as CEA, have been reported (Sorbye and Dahl, 2003; Mundle et al., 2013). Another limitation is that neither methods can provide genetic information describing the intrinsic characteristics of each tumor. Therefore, it is necessary to find a more accurate and minimally invasive biomarker to monitor tumor progression on a frequent basis.

Circulating tumor DNA (ctDNA) can be obtained less invasively than tumor biopsies. Quantification of ctDNA levels and somatic mutations provide real-time information of the highly dynamic tumor characteristics (Diehl et al., 2008). Several studies have suggested that postoperative ctDNA detection helps identify CRC patients who are at a high risk of relapse (Kidess et al., 2015; Reinert et al., 2016; Zhou et al., 2016; Ng et al., 2017). ctDNA has also been proved useful in studying the molecular mechanism for secondary resistance to anti-epidermal growth factor receptor (EGFR) treatment (Morelli et al., 2015; Siravegna et al., 2015; Takegawa et al., 2016). Recently, several investigations reported that early changes of ctDNA are associated with treatment responses in mCRC patients (Tie et al., 2015; Garlan et al., 2017; Thomsen et al., 2018) However, in these studies, candidate targets were selected based on mutations detected in tumor tissues at the time of initial diagnosis, which might be unreasonable due to cancers’ spatial and temporal heterogeneity. Furthermore, two of the three studies focused on ctDNA levels during the first one or two cycles of treatments (Tie et al., 2015; Garlan et al., 2017), and whether ctDNA changes in later cycles can improve the predictive accuracy of long-term prognosi*s* is worth further exploration.

To study the predictive value of ctDNA changes before radiological assessments, we conducted a prospective study that screened mutations in a panel of 50 cancer-related genes in plasma ctDNA collected at multiple time points that covered pre-treatment and each of the four cycles of the first-line systematic treatment of mCRC. Our analysis showed that early changes in ctDNA levels detected via targeted sequencing might potentially predict future treatment responses in mCRCs.

## Materials and Methods

### Patients and Sample Collection

This prospective study recruited patients with mCRC who were treated at Peking Union Medical College Hospital between 2015 and 2017. CRC was pathologically confirmed in eligible patients, and they were set to receive standard first-line chemotherapy using FOLFOX or FOLFIRI, with or without targeted therapy every two weeks. Treatment continued until the establishment of PD or until the completion of 12 cycles of treatment followed by maintenance with capecitabine when the disease was considered PR or SD.

Serial blood samples were collected at the following four defined time-points: pretreatment (within seven days before commencing cycle 1 treatment, named C0) and prior to cycles 2, 3, and 4 of treatments (within three days before commencing the next cycle of treatment, named C1, C2, and C3, respectively). At each time point, traditional serum tumor markers (CEA and CA19-9) were measured and ctDNA analysis was performed. CT scans of the chest, abdomen, and pelvis were performed at baseline and after four cycles of therapy (usually 8–10 weeks after starting treatment). These scans were assessed by a single radiologist, and disease status was evaluated as CR, PR, SD, or PD according to RECIST version1.1.

The study was approved by the human research ethics committee of PUMCH, and all patients provided written informed consent.

### Sample DNA Preparation

Peripheral blood was collected in ethylenediaminetetraacetic acid (EDTA) tubes and centrifuged for 10 min at 1,600 × *g* at 4°C within 2 h of collection. The cell pellets containing PBLs were stored at −20°C until further use. The supernatants were further centrifuged at 16,000 × *g* for 10 min, and plasma was harvested and stored at −80°C until needed. DNA was extracted from PBLs using the E.Z.N.A. Blood DNA kit (Omega Bio-Tek, Norcross, GA, United States), and ctDNA was extracted from plasma using the QIAamp Circulating Nucleic Acid kit (QIAGEN, Hilden, Germany) following the manufacturers’ instructions, respectively. DNA was quantified with the Qubit 2.0 Fluorometer and the Qubit dsDNA HS Assay kit (Thermo Fisher Scientific, Waltham, MA, United States) according to the recommended protocol.

### Ion PGM Library Preparation and Sequencing

The Ion Proton library and DNA sequencing were generated with the Ion AmpliSeq Library Kit 2.0 (Thermo Fisher Scientific, Waltham, MA, United States) according to the manufacturer’s instructions. We used the SV-CA50-ctDNA panel (San Valley Biotech Inc., Beijing, China), which is capable of detecting somatic mutations from plasma samples in 50 cancer-related genes (Guo et al., 2016). Each sample was sequenced with a minimum depth of 10,000×.

### Circulating Tumor DNA Analysis

To determine the minimum variant frequency threshold, the Standard HD780 (Horizon Discovery, Cambridge, United Kingdom) with 100% KRAS G12D, NRAS Q61K, NRAS A59T, and PIK3CA E545K mutations were used as positive controls, and the negative control was wild-type Standards of these positions. These Standards were mixed in 0, 0.5, and 1% proportions of mutation reference standards. Every proportion of reference standard was sequenced 10 times with 10,000× sequencing depth. Supplementary Figure S1 showed the detected mutation frequencies for each of the reference samples. We determined that the detection limit of our sequencing method was 0.5%, which corresponded to a complete separation of positive controls and negative controls.

Initial data from the PGM runs was processed with the Ion PGM platform-specific pipeline software Torrent Suite to generate sequence reads, trim adapter sequences, and filter and remove poor signal-profile reads. Variant calling from the sequencing data was initially generated using Torrent Suite Software v5.0 with a plug-in “variant caller v5.0” program. Four filtering steps were then used to eliminate erroneous base calling and generate final variant calling (Guo et al., 2016). For the first filter, the following parameters were defined for plasma ctDNA: average total coverage depth > 10,000; each variant coverage > 10; a variant frequency of each sample > 0.5%; and *p*-value < 0.01. The second filtering step utilized Integrative Genomics Viewer (IGV) software^1^ or Samtools software^2^ to eliminate possible DNA strand-specific errors after a visual examination of the called mutations. The third filtering step set variants within the 2,855 mutational hotspots according to the manufacturer’s instructions. The final filtering step involved comparing the PBLs to eliminate germ-line mutations.

All ctDNA analyses were performed by individuals blinded to the results of CEA, Ca19-9 and radiological responses. ctDNA levels were quantified as the fraction of mutant alleles.

### Statistical Analysis

Descriptive statistics were used to assess the clinical variables and the frequencies of mutations in pretreatment ctDNA. For each patient, the mutation of the maximal frequency in the pre-treatment plasma ctDNA sample was selected as the candidate target for analysis. We used the log2 fold-change as the indicators of changes in ctDNA, CEA, and CA19-9 levels after each cycle of treatment. For example, ctDNA log2 (C1/C0) refers to the change in the ctDNA level after the first cycle of treatment, with C0 and C1 as the ctDNA levels prior to cycle 1 and cycle 2, respectively. Patient clinical status determined by imaging diagnoses at the end of the fourth treatment cycle was used as the treatment responses. The Mann–Whitney U-test was used to assess the differences in the changes in ctDNA, CEA, and CA19-9 between groups of patients with different responses (i.e., PD vs. non-PD). ROC curves and the *Z* test for the AUC were used to determine the predictive ability of blood biomarkers (changes in ctDNA, CEA, and CA19-9 levels after treatment) for differentiating patients with PD and patients with non-PD at the end of the fourth cycle. The cutoff values were estimated at various sensitivities and specificities and were determined at the maximum Youden’s index. ROC curves were compared with the method of DeLong et al. Survival curves were drawn with the Kaplan–Meier method and compared with the log-rank test. Progression-free survival (PFS) was defined as the time elapsed from the first cycle of treatment until the date of first progression or death (all causes).

The statistical analyses were performed using SPSS Statistics version 19 (IBM Corp), GraphPad Prism version 6.01 (GraphPad Software, Inc., CA), and MedCalc Statistical Software version 15.2 (MedCalc software bvba, Ostend, Belgium). A two-tailed *P* value less than 0.05 was considered statistically significant.

## Results

### Patient Characteristics

**FIGURE 1:**
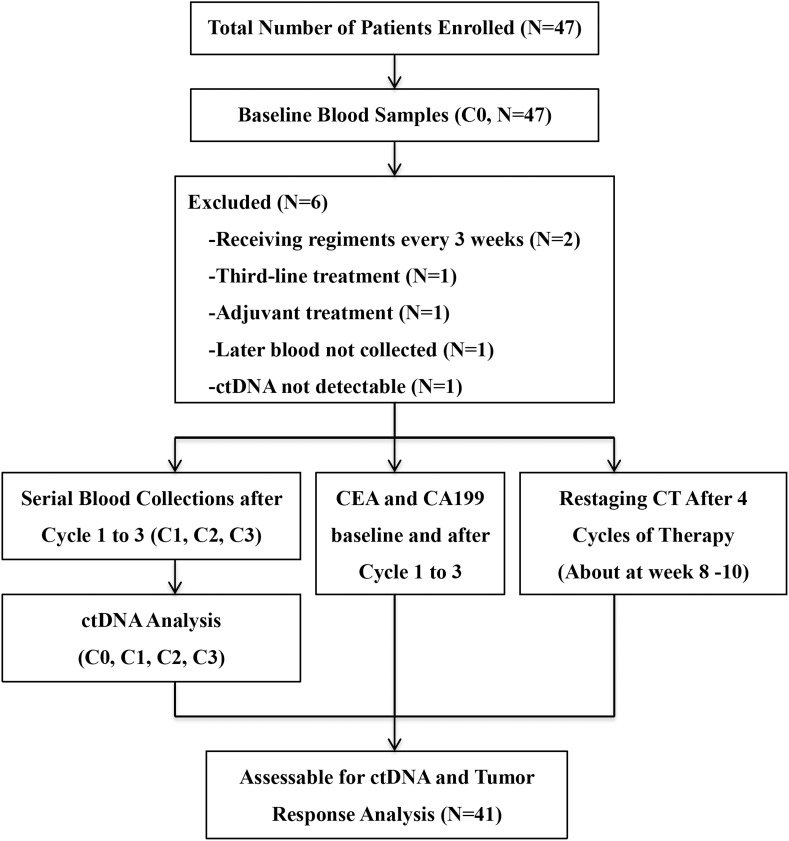
Workflow of the study.

**FIGURE 2:**
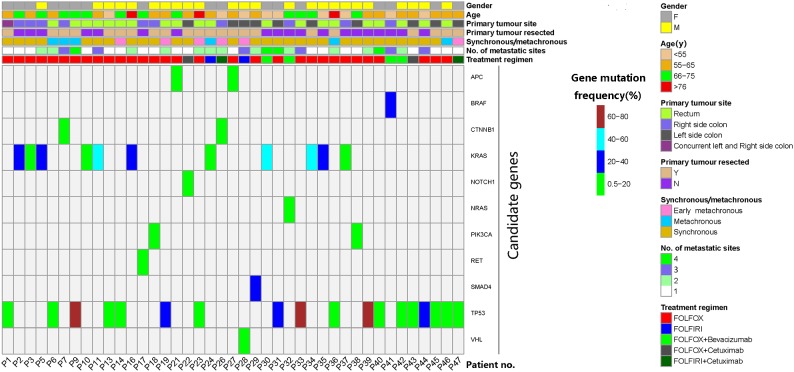
Characteristics and candidate mutations of the 41 patients who were eligible for serial monitoring of circulating tumor DNA (ctDNA). Mutation of the maximal frequency in pretreatment ctDNA was selected as each patient’s candidate mutation. Early metachronous metastasis meant metastasis diagnosed within 12 months of the primary diagnosis.

Between April 2015 and October 2017, 47 patients were enrolled. All patients had at least one baseline blood draw. Mutations in pretreatment (C0) plasma ctDNA were detectable in 45 (95.7%) patients. After excluding four patients who didn’t meet the inclusion criteria, one patient whose later blood samples were not collected and one patient with no detectable ctDNAs, a total of 41 patients entered the study for ctDNA serial monitoring. Figure 1 summarizes the workflow of the study, including the reasons for exclusion from further analysis. The demographic and clinical characteristics of the 41 patients are shown in Figure 2. The median age was 62.0 (28.0–79.0) years old.

### Sequence Coverage Analysis With the Ion PGM

We examined the qualities of PGM reads on sequence lengths, phred scores, GC contents and coverage depths (Supplementary Figure S2). Because reads shorter than 60 bps or longer than 160 bps showed low phred scores and large variations of GC contents, they were removed from further analysis. The remaining 2.5 M reads accounted for 97.64% of the total reads and have an average length of 106 bps, an average GC content of 50% and an average depth of 10,000.

### Pretreatment ctDNA Mutations

The frequencies of mutations in pretreatment ctDNA of the 41 patients are illustrated in Supplementary Figure S3. The three most frequently mutated genes included TP53 (70.73%), KRAS (53.66%), and APC (48.78%). The frequencies of two other commonly studies genes, NRAS and BRAF mutations were 2.44 and 7.32% respectively.

The mutation of the maximal frequency in pre-treatment plasma ctDNA was selected as each patient’s candidate mutation for further analysis. The candidate mutations of the 41 serially monitored patients were illustrated in Figure 2. The median frequency of pretreatment candidate mutations was 7.95% (0.52–76.82%).

### Concordance of RAS Mutation Between Tumor Tissues and Matched Plasma ctDNA Samples

Twenty-five of the 41 patients had results of tumoral RAS status determined by NGS platform-Ion Torrent PGM. Twenty-four tumor tissue samples were from primary tumors, and one sample was from liver metastasis. We used RAS mutations detected in matched tumor tissues and plasma samples to assess the concordance. Based on amino acid substitutions, the concordance of the RAS status between the matched plasma and tissue from each patient was observed in 24 of 25 cases. RAS mutations detected in each sample was summarized in Supplementary Tables S1, S2. Notably, patient No. 1 who was diagnosed with right-side colon cancer with a KRAS G13D mutation and sigmoid colon cancer with a KRAS G12V mutation in tumor tissues suffered from synchronous liver metastases. We detected both KRAS G13D and G12V mutations in her plasma sample (2.05 and 0.92%, respectively, Supplementary Table S2).

### Changes in ctDNA Levels Distinguish Patients With Different Responses Earlier Than Changes in CEA and CA19-9 Levels

**FIGURE 3:**
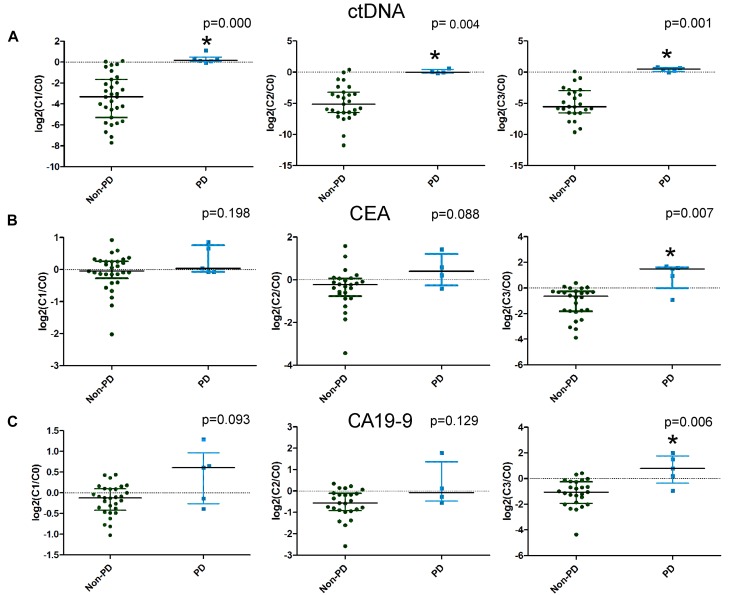
The differences in the changes in circulating tumor DNA (ctDNA), carcinoembryonic antigen (CEA), and carbohydrate antigen 19-9 (CA19-9) levels between progressive disease (PD) and non-PD patients. Changes in ctDNA, CEA, and CA19-9 levels after the first, second, and third cycles of treatment are illustrated in **A–C**, respectively.

Imaging after four cycles of treatment showed 6 PD cases and 35 non-PD cases (17 PR and 18 SD). We compared the changes in the candidate mutations in plasma ctDNA and traditional serum markers after the first (C1), second (C2), and third (C3) cycles of treatment between the non-PD group and the PD group. The ctDNA levels decreased and increased in the non-PD and PD groups, respectively. Significant differences were observed in the changes in the ctDNA levels between the two groups after the first, second, and third cycles (Figure 3A). However, when comparing the changes in CEA and CA199 levels between the two groups, a significant difference was only observed after the third cycle of treatment (Figure 3B,C). These results suggested that changes in ctDNA levels could differentiate patients with PD earlier than changes in CEA and CA199 levels by approximately four weeks.

### Predictive Ability of Blood Biomarkers to Distinguish Patients With PD and Non-PD Responses

Next, we examined the predictive ability of serial changes in ctDNA, CEA, and CA19-9 to identify patients with different responses. Table 1 shows the results of the ROC curve analysis, including the AUC, *P* value, and optimal cutoff points, for the above blood biomarkers to distinguish patients with PD from those with non-PD. As illustrated in Table 1, all the changes in ctDNA between C0 and C1, C2 and C3 were significantly associated with radiologic responses, and there were no statistical differences between the areas under the three ROC curves. For CEA and CA19-9, only changes from pretreatment to immediately before cycle 4 had the potential to predict disease progression.

**Table 1:** Predictive values of the changes in ctDNA, CEA, and CA19-9 levels for the imaging responses of progression disease.

Variable	AUC	*p* value	Cutoff value	Sensitivity (%) (95% CI)	Specificity (%) (95% CI)	PPV (%) (95% CI)	NPV (%) (95% CI)	Accuracy (%) (95% CI)
ctDNA log2 (C1/C0)	0.978	0.000	−0.126	100.0 (54.1–100.0)	93.5 (78.6–99.2)	75.0 (34.9–96.8)	100.0 (88.1–100.0)	94.6 (81.8–99.3)
ctDNA log2 (C2/C0)	0.954	0.004	−0.655	100.0 (39.8–100.0)	92.6 (75.7–99.1)	66.7 (22.3–95.7)	100.0 (86.3–100.0)	93.5 (78.6–99.2)
ctDNA log2 (C3/C0)	0.992	0.001	−0.471	100.0 (47.8–100.0)	96.0 (79.7–99.9)	83.3 (35.9–99.6)	100.0 (85.8–100.0)	96.7 (82.8–99.9)
CEA log2 (C1/C0)	0.683	0.198	−0.079	100.0 (47.8–100.0)	48.3 (29.5–67.5)	25.0 (8.7–49.1)	100.0 (76.8–100.0)	55.9 (37.9–72.8)
CEA log2 (C2/C0)	0.770	0.088	0.158	75.0 (19.4–99.4)	84.0 (63.9–95.5)	42.9 (9.9–81.6)	95.5 (77.2–99.9)	82.8 (64.2–94.2)
CEA log2 (C3/C0)	0.885	0.007	0.666	80.0 (28.4–99.5)	100.0 (86.8–100.0)	100.0 (39.8–100.0)	96.3 (81.0–99.9)	96.8 (83.3–99.9)
CA19-9 log2 (C1/C0)	0.738	0.093	0.523	60.0 (14.6–94.7)	100.0 (88.1–100.0)	100.0 (29.2–100.0)	93.5 (78.6–99.2)	94.1 (80.3–99.3)
CA19-9 log2 (C2/C0)	0.740	0.129	−0.556	100.0 (39.8–100.0)	52.0 (31.3–72.2)	25.0 (7.3–52.4)	100.0 (75.3–100.0)	58.6 (38.9–76.5)
CA19-9 log2 (C3/C0)	0.896	0.006	0.097	80.0 (28.4–99.5)	92.0 (74.0–99.0)	66.7 (22.3–95.7)	95.8 (78.9–99.9)	90.0 (73.5–97.9)

*CEA, carcinoembryonic antigen; CA19-9, carbohydrate antigen 19-9; AUC, area under the curve; PPV, positive predictive value; NPV, negative predictive value; C1/C0, C2/C0, C3/C0, fold-change of levels after cycle 1, cycle 2 and cycle 3, respectively.*

### Prognostic Significance of the Changes in ctDNA Between C0 and C1 for Progression-Free Survival

**FIGURE 4:**
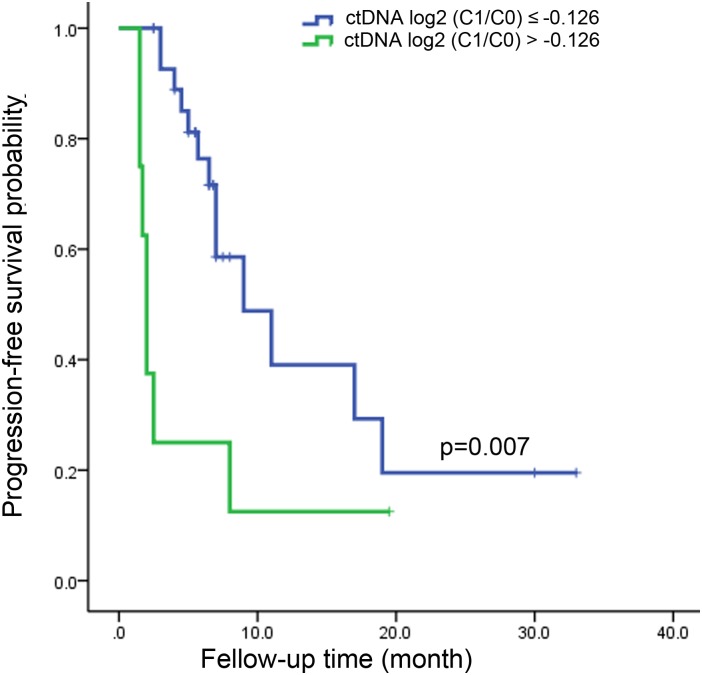
Kaplan-Meier estimate for progression-free survival in metastatic colorectal cancer patients with ctDNA log2 (C1/C0) ≤ –0.126 and ctDNA log2 (C1/C0) > –0.126 after the first cycle of treatment.

From the above analysis, it could be seen that the changes of ctDNA level from C0 to as early as C1 had a certain predictive value of treatment response. The optimal criteria for predicting PD after cycle 4, as determined by the ROC curve, was ctDNA log2 (C1/C0) > −0.126 (Table 1). Patients who met this criterion experienced significantly worse PFS than those with log2 (C1/C0) values below the cutoff point (median PFS, 2.0 v 9.0 months; *P* = 0.007; Figure 4). Patients who met this criterion also experienced significantly worse progression-free survival after the second cycle of treatment (Supplementary Figure S4).

## Discussion

The goal of the present exploratory study was to assess the potential role of the changes in ctDNA levels analyzed via NGS in the early prediction of therapeutic efficacy in first-line-treated mCRC. The results suggested that ctDNA was detectable in a high proportion of systemic treatment-naïve mCRC patients and that early changes in ctDNA was a potential biomarker to predict later radiologically assessed responses to mCRC treatment.

Targeted sequencing via NGS can identify hundreds of mutations starting from a low DNA input (Normanno et al., 2013). Plasma ctDNA represents a minimally invasive source of tumor DNA for molecular profiling and has been shown to be a reliable surrogate for genomic alterations in tumor tissue (Diehl et al., 2008; Bettegowda et al., 2014). Our study demonstrated that mutations in ctDNA could be detected via NGS in 95.7% of systemic treatment-naïve mCRC cases. For the mutation frequencies in plasma, the most commonly mutated ctDNA genes were TP53, KRAS, and APC, which is consistent with mutation profiling in tumor tissues or cell-free DNA in patients with CRC (Cancer Genome Atlas Network, 2012; Strickler et al., 2018). The frequencies of RAS and BRAF mutations in mCRC tumor tissues or ctDNA are reported to be around 50% and 5–10%, respectively (Van Cutsem et al., 2016; Gong et al., 2017; Strickler et al., 2018). Our frequencies of RAS and BRAF mutations in ctDNA of mCRC were similar to those reported in the literature.

We also confirmed the high concordance of ctDNA and tumor tissues for RAS status. Many previous studies have demonstrated the concordance of ctDNA and tDNA for the molecular characterization of RAS and BRAF. Thierry et al. (2014) found that ctDNA had a sensitivity of 92%, a specificity of 98%, and a net accuracy of 96% for KRAS mutations in mCRC patients. The prospective trial of Bachet et al. (2018) enrolled 425 mCRC patients to compare plasma with tissue RAS analysis, and results demonstrated 94.8% accuracy in 329 patients with detectable ctDNA. Some researchers believe that ctDNA represents the average tumor genome and may be a more accurate approach for mutation identification (Sartore-Bianchi et al., 2016a). In our study, ctDNA had a sensitivity of 93.3%, a specificity of 100%, and a net accuracy of 96.0% for RAS mutations compared with tumor tissue, which are comparable to the results of the above studies.

The ability to perform non-invasive dynamic monitoring for tumors has been a challenge for clinicians for many years. ctDNA carries information on the tumor burden and has emerged as a good candidate for tracking tumor dynamics in different cancers, potentially circumventing the need for repeated tumor biopsies. ctDNA measurements could be used to reliably monitor tumor dynamics in subjects with CRC who are undergoing surgery or chemotherapy (Diehl et al., 2008). The half-life of ctDNA is less than 2 h (Diaz and Bardelli, 2014). ctDNA responds promptly to treatments, and changes in its levels could be evident days or weeks before tumor shrinkage can be assessed by imaging or the emergence of obvious changes in traditional serum tumor biomarkers. Serial profiling of ctDNA holds immense promise for early and accurate detection of patient responses. For example, in the studies of metastatic prostate cancer (Goodall et al., 2017) and breast cancer (O’Leary et al., 2018), the results suggested that early dynamic changes in ctDNA might be biomarkers for drug efficacy prediction.

In metastatic CRC, there are a few previous studies focusing on the value of early change in ctDNA for predicting therapeutic responses (Tie et al., 2015; Garlan et al., 2017; Thomsen et al., 2018). Tie et al. (2015) and Garlan et al. (2017) chose the candidate mutations in ctDNA based on the mutations in tumor tissue. Thomsen et al. (2018) only included patients with RAS/RAF mutations and monitored dynamic changes in circulating RAS/RAF mutations. Tie et al. found that a ten-fold reduction of ctDNA after one cycle of therapy was associated with good response, and Garlan et al. used slope of changes as the marker of enriched response rates. Thomsen et al. suggested that a low level of RAS/RAF mutations after the first cycle was associated with a low risk of progression. Although all the above results suggested that early dynamics in ctDNA was associated with treatment responses, the research methods were different and how to exploit this issue in the clinic remains unclear. Unlike these studies, our investigation screened candidate mutations for further analysis directly in baseline plasma by an NGS high-throughput platform, which could reflect the real-time situation of the biological characteristics of tumors more accurately than monitoring candidate mutations chosen based on tissue mutations at initial diagnosis. Furthermore, we analyzed the changes in ctDNA, CEA, and CA19-9 after every cycle of treatment before the first imaging evaluation. Later samples could have added value. We compared the predictive ability of ctDNA with conventional serum tumor markers in the prediction of therapeutic response after each cycle of treatment. Our results showed that changes in ctDNA could differentiate patients with responses of PD earlier than changes in traditional serum tumor markers by approximately one month. Although the predictive power of changes in ctDNA after cycle 3 was slightly superior to the previous two cycles in data of AUC and accuracy, there was no statistically significant difference. CtDNA reductions as early as prior to cycle 2 predicted the radiologic responses after cycle 4 with an accuracy of 94.6%. Our study also revealed significantly worse PFS in patients with increased ctDNA levels. Thus, our study added to growing evidence that serial ctDNA monitoring showed the potential of early changes in ctDNA to predict later tumor responses.

To the best of our knowledge, there are no reliable clinicopathological factors in the literature that could predict the efficacy of chemotherapy in mCRC so far. We also analyzed the relationship between the clinicopathological factors including histology, chemotherapy regimen, number of metastases, etc., and early progression in this group of patients. However, no clinical or pathological factors predicting responses were found (data not shown). Our results of early changes in ctDNA levels predicting future treatment responses are appealing because detecting disease progression via non-invasive monitoring and convenient markers is important in clinical practice to avoid continuing ineffective therapies and prevent unnecessary side effects. Nevertheless, our study has some limitations. This is a single-center exploratory study with a small sample size. Our data are insufficient to determine the optimal criteria for assessing the ctDNA concentration after treatment, and larger cohorts of patients are needed to draw robust conclusions and to compare the impact on progression-free or overall survival of changes in ctDNA levels. Although ctDNA helps detect disease progression earlier, imaging is still necessary to determine the anatomy and the respectability of tumor lesions. Therefore, it is unlikely that ctDNA would completely replace imaging in assessments of tumor burden. However, ctDNA analysis allows a more comprehensive assessment of tumors, and the detection of ctDNA is one of the most promising approaches for improving tumor monitoring procedures in mCRC. Furthermore, if this method would be included in the clinical routine, it might alter the timing of the imaging and change the exposure of patients to inefficient treatment.

In summary, our data suggests that ctDNA mutations could be detected in a high proportion of treatment-naïve mCRC patients via NGS. Early changes in the ctDNA levels showed the potential to predict later radiologic responses, and ctDNA monitoring might be integrated with imaging to assess responses to anticancer treatment in mCRC.

## Ethics Statement

This study was carried out in accordance with the recommendations of the human research ethics committee of Peking Union Medical College Hospital (PUMCH) with written informed consent from all subjects. All subjects gave written informed consent in accordance with the Declaration of Helsinki. The protocol was approved by the human research ethics committee of PUMCH.

## Author Contributions

CB and JZ conceived and designed the study. YZ, JZ, NJ, YC, and ZS contributed to sample collections. CS, WC, XW, and RS performed ctDNA sequencing and analysis. XG assessed the imaging responses. JZ, NJ, ZS, and CB analyzed and interpreted the data with the help of NL and XW. JZ and NJ wrote the manuscript with the help of XW, ZS, and CB. All the authors read and approved the final manuscript.

## Conflict of Interest Statement

CS, WC, XW, and RS are employees of San Valley Biotechnology, Inc., in Beijing, China. The remaining authors declare that the research was conducted in the absence of any commercial or financial relationships that could be construed as a potential conflict of interest.

## Footnotes

1 http://www.broadinstitute.org/igv

2 http://samtools.sourceforge.net

## Abbreviations

AUC: area under curve
CA19-9: carbohydrate antigen 19-9
CEA: carcinoembryonic antigen
CR: complete response
CRC: colorectal cancer
CT: computed tomography
ctDNA: circulating tumor DNA
mCRC: metastatic colorectal cancer
NGS: next-generation sequencing
PBLs: peripheral blood lymphocytes
PD: progressive disease
PR: partial response
RECIST: Response Evaluation Criteria in Solid Tumors
ROC: receiver operating characteristic
SD: stable disease
tDNA: tissue DNA.
