# Supplementary figures and images for: Serial Monitoring of Circulating Tumor DNA in Patients With Metastatic Colorectal Cancer to Predict the Therapeutic Response
Source: Front Genet. 2019 May 21;10:470. doi: 10.3389/fgene.2019.00470 (PMC6536571; doi:10.3389/fgene.2019.00470)

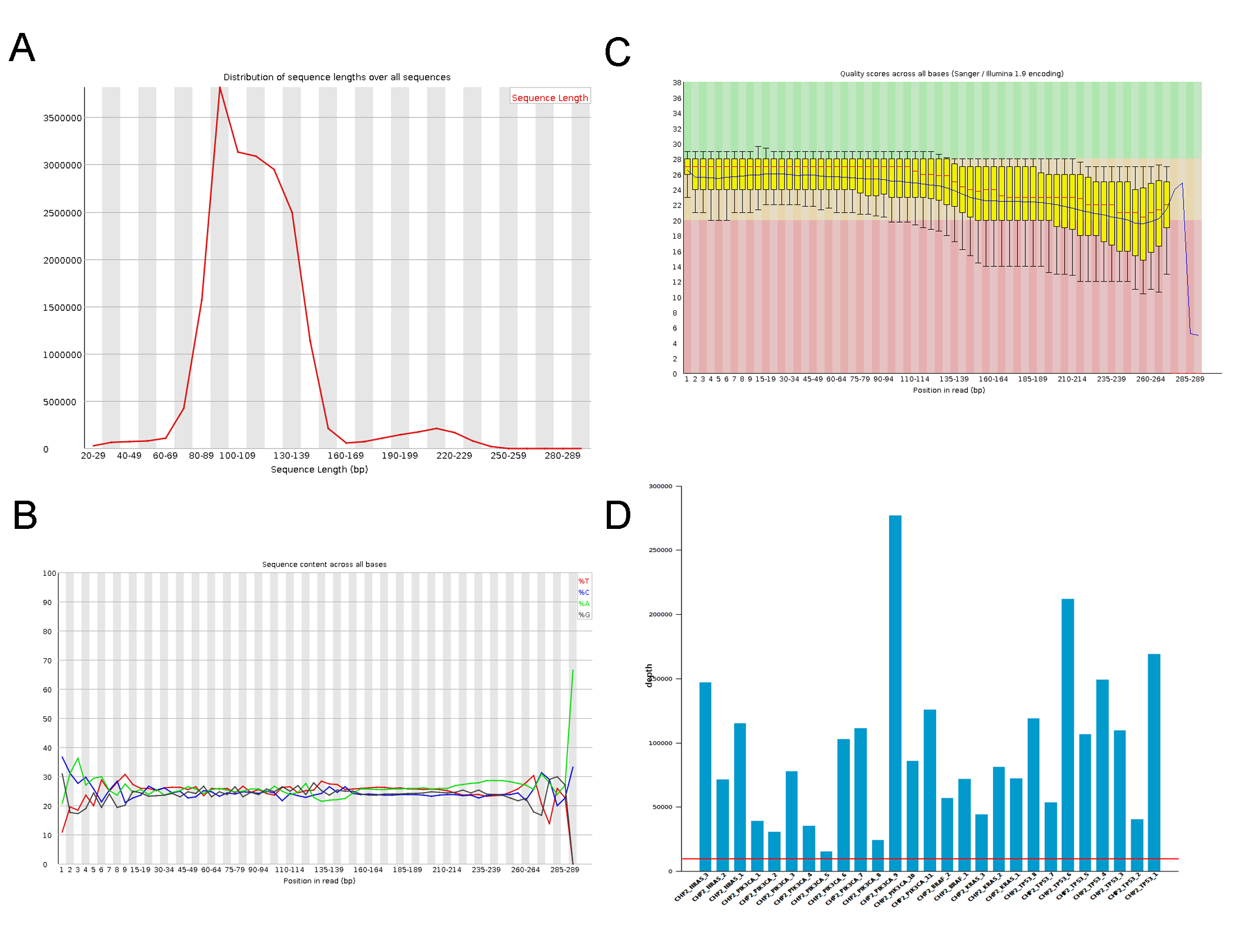

Supplement: Supplementary file 2 [file Image_2.TIF]

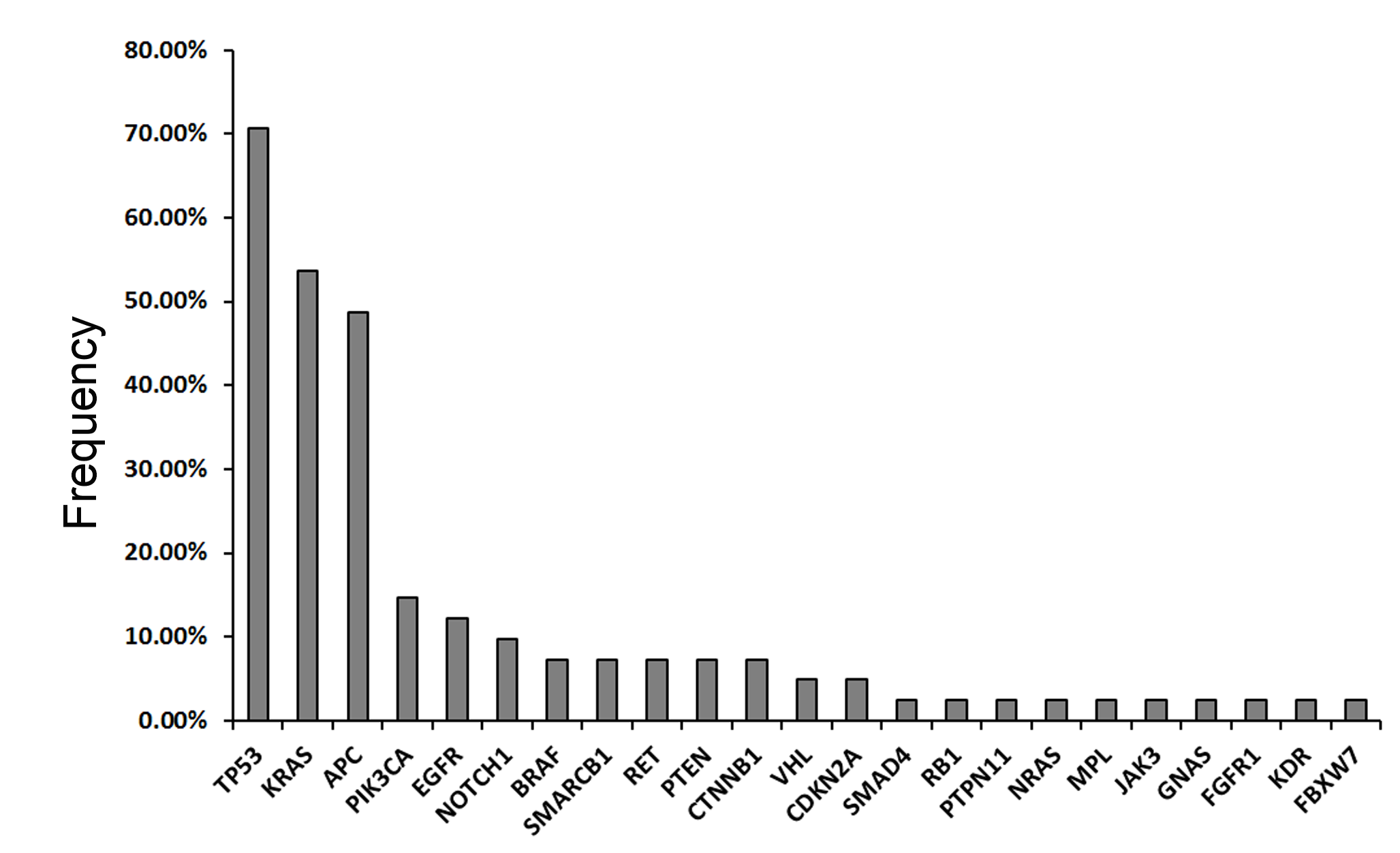

Supplement: Supplementary file 3 [file Image_3.TIF]

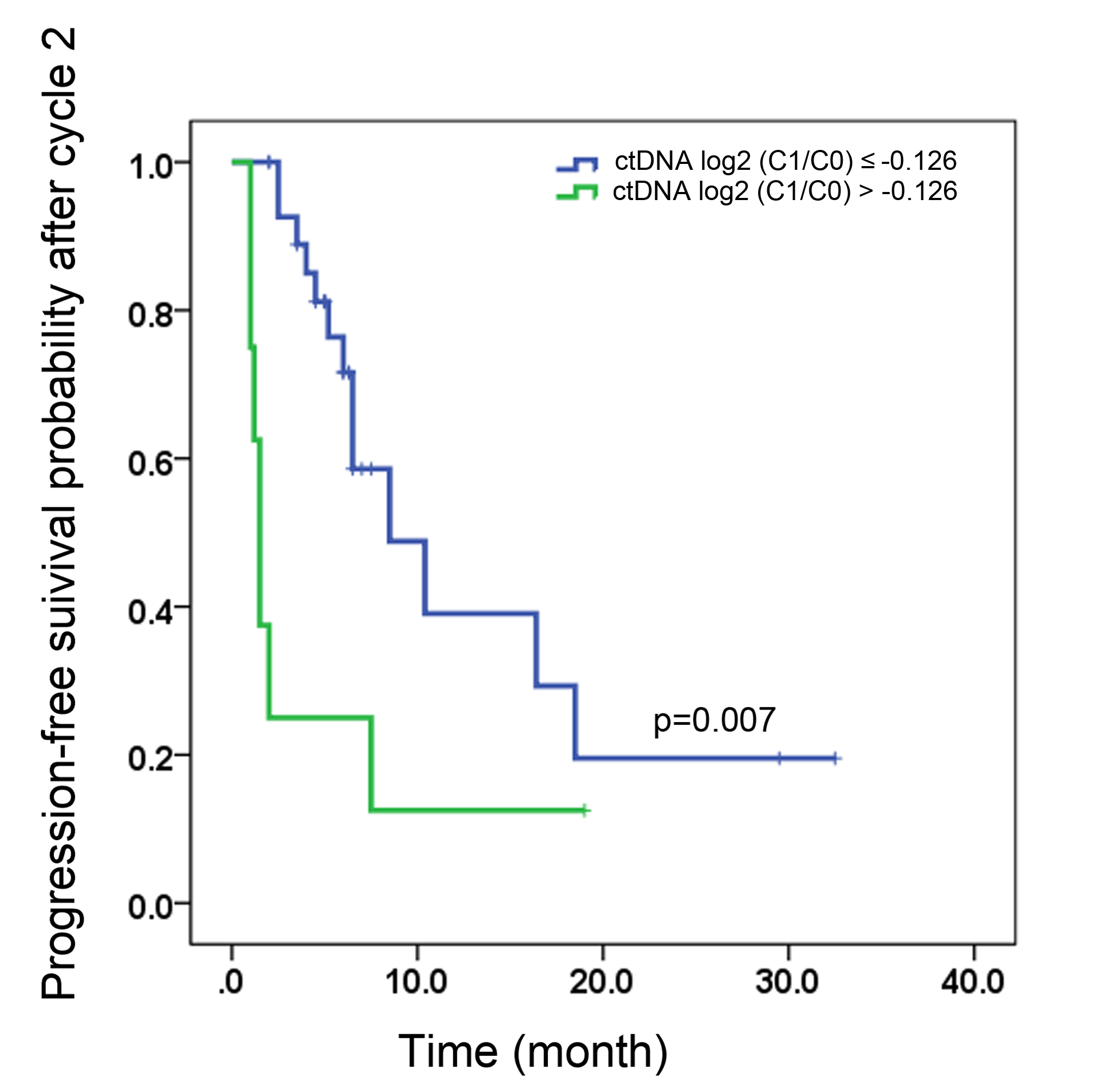

Supplement: Supplementary file 4 [file Image_4.TIF]
