# Supplementary material for: Serial Monitoring of Circulating Tumor DNA in Patients With Metastatic Colorectal Cancer to Predict the Therapeutic Response
Source: Front Genet. 2019 May 21;10:470. doi: 10.3389/fgene.2019.00470 (PMC6536571; doi:10.3389/fgene.2019.00470)
Supplement: Supplementary file 5 [file Table_1.DOCX]

Supplementary Table S1. Concordance of plasma and tissue RAS mutation results

|  |  | Tumour tissue RAS result | | |
| --- | --- | --- | --- | --- |
|  | RAS | Mutant | Wild | Total |
| Plasma ctDNA RAS result | Mutant | 14 | 0 | 14 |
|  | Wild | 1 | 10 | 11 |
|  | Total | 15 | 10 | 25 |
| Sensitivity | 93.33% |  |  |  |
| Specificity | 100.00% |  |  |  |
| PPV | 100.00% |  |  |  |
| NPV | 90.91% |  |  |  |
| Concordance | 96.00% |  |  |  |

Abbreviation: PPA: positive percent agreement; NPA: negative percent agreement.
