# Supplementary material for: Serial Monitoring of Circulating Tumor DNA in Patients With Metastatic Colorectal Cancer to Predict the Therapeutic Response
Source: Front Genet. 2019 May 21;10:470. doi: 10.3389/fgene.2019.00470 (PMC6536571; doi:10.3389/fgene.2019.00470)
Supplement: Supplementary file 6 [file Table_2.DOCX]

Supplementary Table S2. Mutations and mutation frequencies of RAS and BRAF identified in paired tDNA and plasma ctDNA

| Patient no. | Gene | Mutation type | AA mutation | % mutation in PBL (reads) | % mutation in ctDNA (reads) | % mutation in tDNA (reads) |
| --- | --- | --- | --- | --- | --- | --- |
| 1 | KRAS | SNV | p.G13D | 0.049 (2031) | 2.054 (23950) | 15.748 (127) |
|  | KRAS | SNV | p.G12V | 0.000 (2023) | 0.924 (23925) | 18.056 (144) |
| 2 | KRAS | SNV | p.G13D | 0.026 (3779) | 23.182 (29122) | 27.138 (947) |
| 5 | KRAS | SNV | p.G13D | 0.043 (2325) | 24.333 (15810) | 31.231 (1332) |
| 6△ | — | — | — | — | — | — |
| 11 | KRAS | SNV | p.G12D | 1.145 (262) | 41.776 (9120) | 7.576 (990) |
| 13△ | — | — | — | — | — | — |
| 17* | KRAS | SNV | p.G12D | 0.087 (2308) | 0.013 (15085) | 30.788 (812) |
| 19 | KRAS | SNV | p.G12D | 0.000 (2603) | 24.330 (4217) | 24.236 (2389) |
| 21△ | — | — | — | — | — | — |
| 22△ | — | — | — | — | — | — |
| 23 | KRAS | SNV | p.G12A | 0.000 (5804) | 0.740 (13656) | 51.397 (1002) |
| 26△ | — | — | — | — | — | — |
| 27△ | — | — | — | — | — | — |
| 29 | KRAS | SNV | p.G12A | 0.000 (12652) | 21.057 (20369) | 11.935 (3519) |
| 30 | KRAS | SNV | p.G13D | 0.082 (19567) | 50.191 (29105) | 55.892 (2215) |
| 32 | NRAS | SNV | p.Q61R | 0.056 (7151) | 1.457 (40756) | 12.006 (1366) |
| 34 | KRAS | SNV | p.G13D | 0.203 (3441) | 48.669 (26563) | 22.19 (1397) |
| 35 | KRAS | SNV | p.G12D | 0.023 (13271) | 22.257 (23381) | 13.546 (1351) |
| 36△ | — | — | — | — | — | — |
| 37 | KRAS | SNV | p.G12D | 0.000 (3146) | 0.518 (14853) | 21.792 (2299) |
| 38 | KRAS | SNV | p.G12D | 0.000 (1586) | 1.3 (18152) | 59.833 (2873) |
| 39 | KRAS | SNV | p.G12D | 0.059 (6798) | 37.603 (13467) | 23.930 (3552) |
| 40 | BRAF | SNV | p.D594N | 0.046 (4316) | 6.391 (10029) | 18.511 (2928) |
| 42△ | — | — | — | — | — | — |
| 43△ | — | — | — | — | — | — |

△: Patients’ RAS or BRAF were wild type in both tissue DNA and plasma ctDNA. *: RAS was mutant type in tDNA and wild type in ctDNA in patient No. 17.
