# Supplementary material for: Serial Monitoring of Circulating Tumor DNA in Patients With Metastatic Colorectal Cancer to Predict the Therapeutic Response
Source: Front Genet. 2019 May 21;10:470. doi: 10.3389/fgene.2019.00470 (PMC6536571; doi:10.3389/fgene.2019.00470)
Supplement: Supplementary file 7 [file Presentation_1.pdf]

Supplementary Figure S1. Detection limit of the sequencing method. The Standard HD780 (Horizon Discovery, Cambridge, UK) with 100% KRAS G12D, NRAS Q61K, NRAS A59T, and PIK3CA E545K mutations were used as positive controls, and the negative control was wild-type Standards of these positions. These Standards were mixed in 0, 0.5, and 1% proportions of mutation reference standards. Every proportion of reference standard was sequenced 10 times with 10,000x sequencing depth. The results illustrate that the detection limit of our sequencing method is 0.5%.

Supplementary Figure S2. Sequence coverage with the Ion PGM. (A) The distribution of the sequence lengths over all ctDNA (plasma) sequences is shown. The distribution of sequence lengths was essentially normal, and most of the lengths were between 60 and 160 bp. Therefore, the sequences within this length range were selected for further analysis. (B) The GC content across all bases of ctDNA (plasma) was approximately 50%. However, the GC content from 1 to 20 bp fluctuated widely. Therefore, this region of reads was removed during quality control. (C) The quality scores across all bases of ctDNA (plasma). Every base called from next-generation sequencing has a quality score, illustrating the accuracy of sequencing. (D) The depth of most amplicons was over 10,000x, and the average base coverage depth of the plasma samples was greater than 10,000x.

Supplementary Figure S3. The frequencies of mutations in the pretreatment (C0) plasma ctDNA of the 41 serially monitored patients.

Supplementary Figure S4. Kaplan-Meier estimate for progression-free survival after the second cycle of treatment in metastatic colorectal cancer patients with ctDNA log2 (C1/C0) ≤ -0.126 and ctDNA log2 (C1/C0) > -0.126 (median PFS, 8.5 v 1.5 months; *P* = 0.007).
